# The effect of Phytosphingosine and bioactive glass-ceramics in preventing dental enamel erosion

**DOI:** 10.1590/0103-6440202304904

**Published:** 2023-05-15

**Authors:** Leticia Campos de Araujo, Ayodele Alves Amorim, Rocio Geng Vivanco, Carolina Noronha Ferraz de Arruda, Floris J Bikker, Fernanda de Carvalho Panzeri Pires-de-Souza

**Affiliations:** 1 Department of Dental Materials and Prosthodontics, Ribeirão Preto School of Dentistry, University of São Paulo, Ribeirão Preto, SP, Brazil.; 2 Department of Periodontology and Oral Biochemistry, Academic Centre for Dentistry Amsterdam (ACTA), Vrije Universiteit and Universiteit van Amsterdam, Amsterdam, The Netherlands.

**Keywords:** Bioactive glass-ceramic, Color stability, Erosive challenge, Microhardness, Phytosphingosine

## Abstract

This study evaluated the effect of phytosphingosine (PHS) and bioactive glass-ceramic (Biosilicate) on dental enamel in terms of color alteration (ΔE), microhardness, and surface roughness when submitted to erosive challenge (EC). Sixty specimens of bovine teeth (6×6×2mm) were obtained. Initial color (Easyshade, VITA), KHN (HMV-2, Shimadzu), and Ra (SJ-201P, Mitutoyo) measurements were performed. Specimens were separated into groups according to treatments: PHS, 10% Biosilicate, PHS+10% Biosilicate, and artificial saliva (control) and submitted to EC with Coca-Cola for 2 min. This cycle was repeated 4 times daily/15 days. Between cycles, specimens remained in artificial saliva (2 h/37°C). After daily cycles, they were also stored in artificial saliva at 37ºC. Final color, microhardness, and surface roughness measurements were done. Color and KHN data were analyzed by one-way ANOVA, Tukey’s test; and Ra, by 2-way ANOVA, repeated measures, and Tukey’s test (p<.05). The highest ΔE occurred in Saliva+EC (p<.05). Groups treated with PHS presented lower color change than Saliva+EC (p<.05). All the groups presented mean values above the 50:50% perceptibility (50:50%PT) and acceptability (50:50%AT) thresholds, except for control that showed mean value above 50:50%PT but below 50:50%AT. Biosilicate+EC showed higher relative microhardness than Saliva+EC (p<.05), but was similar to PHS+EC and PHS+Biosilicate+EC. Final enamel surface roughness increased for all the groups (p<.05), except for the control. The Biosilicate may prevent enamel mineral loss induced by erosion better than saliva. The PHS associated or not to Biosilicate demonstrated better color stability than saliva.

## Resumo

Este estudo avaliou o efeito da Fitoesfingosina (PHS) e da vitrocerâmica bioativa (Biosilicato) sobre o esmalte dental em termos de alteração de cor (ΔE), microdureza (KHN) e rugosidade superficial, quando submetido a desafio erosivo (DE). Sessenta espécimes de dentes bovinos (6×6×2mm) foram obtidos. Foram realizadas leituras de cor inicial (Easyshade, VITA), microdureza (HMV-2, Shimadzu) e rugosidade superfícial (SJ-201P, Mitutoyo). Os espécimes foram separados em grupos de acordo com os tratamentos: PHS, Biosilicato a 10%, PHS+Biosilicato a 10%, e saliva artificial (controle). Em seguida, foram submetidos a DE com Coca-Cola por 2 min. Esse ciclo foi repetido 4 vezes/dia por 15 dias. Entre os ciclos, as amostras foram mantidas em saliva artificial (2 h/37°C). Após os ciclos diários, os espécimes também foram armazenados em saliva artificial a 37ºC. Foram realizadas leituras finais de cor, microdureza e rugosidade superficial. Os dados de cor e microdureza foram analisados ​​por ANOVA de uma via, teste de Tukey; e dados de rugosidade superficial, por ANOVA de duas vias, teste de Tukey (p<.05). A maior ΔE ocorreu em Saliva+DE (p<.05). Grupos tratados com PHS apresentaram menor alteração de cor do que Saliva+DE (p<.05). Biosilicate+DE demonstrou valores intermediários, similar (p>.05) aos outros grupos, exceto Saliva+DE. Todos os grupos presentaram média acima dos limites 50:50% de perceptibilidade (50:50%LP) e aceitabilidade (50:50%LA) exceto o controle que demonstrou média acima do 50:50%LA mas abaixo do 50:50%LP. Biosilicate+DE mostrou maior microdureza realativa do que Saliva+DE (p<.05), mas similar a PHS+DE e PHS+Biosilicato+DE. A rugosidade de superfície do esmalte aumentou para todos os grupos, exceto para o controle que presentou a menor alteração (p<.05). O Biosilicato apode prevenir perda mineral do esmalte indizido pela erosão melhor que a saliva. O PHS associado ou não ao Biosilicato demonstrou melhor estabilidade de cor que a saliva.

## Introduction

Dental erosion has become a significant public health concern, highly influenced by changes in habits and lifestyles over time^1^. It occurs due to the chemical dissolution of the tooth enamel^2^, mainly by the acid attack^3^. This condition is being increasingly reported. The literature shows that the prevalence of advanced dental erosion among young adults is increasing by up to 30%^4^.

Dental erosion has a multifactorial etiology, mainly caused by intrinsic (endogenous acids) and extrinsic (exogenous acids) factors^5^. Gastric acids during vomiting or regurgitation, especially in patients with gastroesophageal reflux disease and bulimia nervosa, can contribute to this disease as an intrinsic factor. On the other hand, exogenous acids often come from the diet, environment, medication, and lifestyle^6^.

Among the extrinsic source, the excessive and continuous consumption of soft drinks with low pH (pH < 5.5) is a primary risk factor for the development of this oral health problem^7^ ^,^ ^8^. Recurrent exposure of hard tissues to soft drinks can cause irreversible damage^9^, as dental erosion can alter the micromorphological surface of dental enamel, leading to reduced microhardness^10^, and consequently, tooth wear^10^ ^,^ ^11^. An increase in the surface roughness of the enamel can also induce the absorption of pigments, and consequently, lead to enamel color change^10^.

Human saliva can dilute and clear erosive agents and presents buffering capacity, that could neutralize some acids. In addition, it predominantly contributes to the formation of a salivary protein-based layer called the acquired pellicle that covers the tooth surface, reducing its contact with acids and providing deposition of calcium and phosphate ions^12^. However, saliva provides only partial protection against erosion^13^ ^,^ ^14^ and the intrinsic remineralization induced due to its chemical composition is a slow process that does not provide significant recovery of structure properties of subsurface lesions^15^. 

Dentistry seeks an active substance capable of protecting the tooth enamel from mineral loss and maintaining the dynamics between demineralization/remineralization, especially during daily eating habits. Fluoride-containing formulations, such as sodium fluoride, have been extensively studied as an anti-erosive agent. In high concentrations, they promote the precipitation of a calcium fluoride layer in the enamel that acts as a fluoride reservoir and reduces the dissolution of enamel exposed to acids^16^. The use of calcium silicate and sodium phosphate-based compounds, such as casein phosphopetides and amorphous calcium phosphates, has also been proposed^17^. They release calcium and phosphate that promotes hydroxyapatite precipitation on the tooth surface, increasing the hardness of eroded enamel^17^ ^,^ ^18^. Nonetheless, the protective effect of these agents is still controversial^18^ ^,^ ^19^ and they require an intensive application regime^20^.

These remineralizing agents provide protection against demineralization, preventing dental erosion. Nevertheless, it would be important that, during demineralization-remineralization process, the remineralization agents not only prevent demineralization, but also induce remineralization of the tissue, replacing the lost minerals and not only preventing their loss. Thus, remineralizing agents, such as bioactive glasses, could be an excellent alternative.

The prevalence of dental erosion has considerably increased, thus new and more effective compounds should be investigated^15^. Substances with alternative and multiple mechanisms of action have been studied to optimize the available prevention strategies (21-23). The formation of a protective layer on hard tissue appears to be effective against erosive challenge^9^. Recent studies have demonstrated the protective capacity of phytosphingosine (PHS), one of the main constituents of sphingolipids (lipid molecules found abundantly in tissues of fungi, plants, and animals, including humans)^21^ ^,^ ^22^. It has aliphatic hydrocarbon ramifications combined with positive ending functional groups^21^.

Studies have shown that PHS significantly protects hydroxyapatite prisms against acid-induced tissue loss^3^ ^,^ ^22^. Unlike fluoride, PHS does not change the chemical composition of the enamel^22^. It forms a layer that acts as a barrier against H^+^ ions^3^ ^,^ ^22^ and inhibits bacterial adherence to hydroxyapatite^21^ ^,^ ^22^. Because of these anti-erosive and anti-microbial characteristics, PHS can be considered an interesting ingredient in oral hygiene products to prevent dental erosion.

Other remineralizing therapies such as the application of Biosilicate have been researched^23^ ^,^ ^24^. Biosilicate is a bioactive glass ceramic with crystallized particles, which has shown promising results in the formation of hydroxycarbonapatite on mineralized tissues^23^. Moreover, when in contact with dentin, Biosilicate dissolves, constantly releasing calcium and phosphate ions, raising the pH, and thus, favoring the process of dental remineralization^24^.

The formation of a protective layer with ions attached can protect the tooth surfaces from erosion and/or prevent the progression of the lesions. However, there are few studies evaluating the association of these strategies^25^ and the possible synergistic effect.

Erosive agents cause mineral loss that reduces the surface hardness of the enamel and increases the tooth roughness. The resistance of the substrate to indentation can measure alterations on the enamel hardness. The microhardness analysis is a simple and reliable test to determine dental erosion or the protective effect of treatments on this condition^26^. On the other hand, changes in the surface roughness can indicate early erosion^27^ and this alteration could be evaluated with a roughness meter.

Furthermore, as the enamel wears away and becomes more translucent, the underlying dentin is gradually revealed and the teeth may look yellow^28^. So, color alteration could also be an indicator of enamel erosion. 

Therefore, considering the above, the aim of this study was to evaluate the effect of Phytosphingosine and Biosilicate in the protection of dental enamel, in terms of color stability, microhardness and surface roughness, when submitted to erosive challenge. The null hypothesis tested was that there would be no difference in the color and microhardness of the enamel treated with Phytosphingosine and/or Biosilicate compared with the ones immersed in saliva.

## Materials and methods

### Specimens Preparation

**Figure 1:**
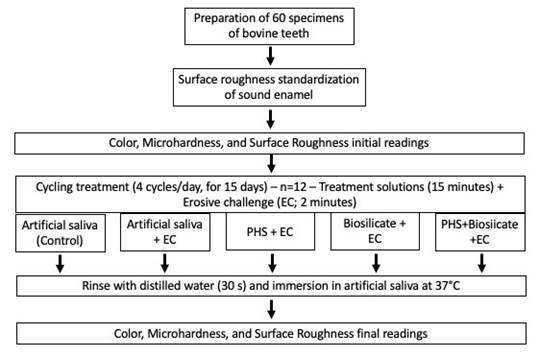
Flow diagram of the study. Treatment and Erosive challenge were performed four times daily for 15 days. PHS, phytosphingosine.

The sample size was calculated based on a pilot study comparing the difference between the mean values of microhardness, requiring at least 12 specimens (power of 80%; α = 0.05) per group (Figure 1). Bovine teeth without cracks, fractures and stains were cut using a low-speed diamond disk (Isomet 1000, Isomet, Buehler, Lake Bluff, IL, USA). to obtain sixty fragments (6 x 6 x 2 mm). The bovine fragments were marked in the middle of the border of the dentin surface using a diamond disc to ensure the same positioning during the tests. The dentin surface was not evaluated in the present study. The initial surface roughness of the enamel was standardized to avoid possible interferences in the results. The enamel surface of each bovine fragment was polished with 600- and 1200-grit sandpaper in descending order, under water cooling. Three readings of the enamel surface roughness were performed using a rugosimeter (Surfcorder SE 1700, Kosakalab, Tokyo, Japan). One in the center (according to the mark made earlier) and two at 1 mm to the left and to the right, respectively. The average of these three measures was considered as the initial surface roughness. Only fragments that presented surface roughness between 0.05 μm and 0.12 μm were included in the study^29^ since the surface roughness has a direct influence on the color^30^.

### pH Cycling Procedure

Before the pH cycling procedure, the dentin of each fragment was protected with hot glue. All the bovine teeth fragments were randomly separated into 5 groups (n = 12) (Figure 1). They were immersed in the treatments as described in Table 1 and then, submitted to Erosive challenge with Coca-Cola (pH = 2,5; The Coca-Cola Company, Atlanta, USA) for 2 minutes^31^. The pH was measured using a digital pH meter (Kasvi model K39-2014B, Paraná, Brazil) calibrated with standard solutions of pH 4.0 and 7.0. After immersion in Coca-Cola, the fragments were rinsed with distilled water. This cycle (Treatment followed by EC) was repeated 4 times daily for 15 days. Between the cycles, the specimens were rinsed with distilled water and remained in artificial saliva for 2h/37ºC. At the end of each day, the fragments were stored in artificial saliva at 37 °C until the next day^31^ ^,^ ^32^. This *in vitro* erosive model simulates the effect of the acidic soft drink on a prolonged erosion. Thus, there would be small amounts of tissue loss over relatively short time periods caused by a single potentially erosive beverage^31^ ^,^ ^32^.

**Table 1:** Treatments

Group	Treatment	Application Protocol
Control	Artificial Saliva	Immersion in 1 mL artificial saliva in a microtube for 15 days.
Saliva+EC	Artificial saliva followed by erosive challenge	Immersion in 1 mL artificial saliva in a microtube for 15 min, then EC
PHS+EC	PHS followed by erosive challenge	Immersion in 1 mL 0,01% PHS solution in a microtube for 15 min under shaking (150 rpm), then EC
Biosilicate+EC	Biosilicate suspension followed by erosive challenge	Immersion in 1 mL 10% Biosilicate in a microtube for 15 min, then EC
PHS+Biosilicate+EC:	Biosilicate suspension diluted in PHS, followed by erosive challenge	Immersion in 1 mL mixture of 10% Biosilicate and 0,01% PHS in a microtube for 15 min under shaking (150 rpm), then EC

Artificial saliva was prepared with 0.1665 g of calcium chloride, 0.133 g of monosodium phosphate, 11.184 g of potassium chloride, 0.02 g of sodium azide, and 2.4228 g of Tris buffer; diluted in 1 liter of deionized water^33^. This solution was stored in the dark.

PHS solution was prepared in ethanol at a concentration of 5 mg/mL and diluted to 100 at 100 µg/mL (0,01%) in 20 mM Tris supplemented with 0.1% Tween 20 (pH = 6.8) (Tris- Tween) to keep the PHS in solution^3^ ^,^ ^22^ ^,^ ^23^. This solution was stored in the dark^3^ ^,^ ^21^.

Biosilicate particles were used at a concentration of 10%^34^. For this, 0.15 mg of Biosilicate was added to 1.35 mL of distilled and deionized water immediately before each immersion.

Biosilicate particles were also mixed into PHS solution. 0.15 mg of Biosilicate was added to 1.35 mL of PHS solution immediately before each immersion.^3^ ^,^ ^21^.

### Color Analysis

Before and after the pH cycle procedure, the color measurements were taken using a spectrophotometer (Vita Easyshade, VITA Zahnfabrik, Bad Sckingen, Germany) on a standard white background (Mast Quality Solutions, Santo André, SP, Brazil). The digital tip of the spectrophotometer has the same size as the specimen (6 mm), ensuring the same reading area for both initial and final measures. The optical geometry of the color measurement simulates a 45º/0º geometry.^33^.

CIELab observation pattern was used, considering color dimensions of black-white luminosity (L*), green-red (a*) and blue-yellow (b*), so that the L* axis is perpendicular to the a* and b*. A visible spectrum of light (400 to 700 nm) is focused on the object, and the reflection is measured. The L*, a*, b* values of each specimen were measured before and after the protocols, and the baseline mean values are presented in Table 2. Color stability was calculated using the CIEDE2000 formula which is based on the Lab coordinates^35^. L*, a*, b* values were recorded in Excel sheets developed by Sharma et al.^35^ and available electronically at https://www.hajim.rochester.edu/ece/sites/gsharma/ciede2000/ that calculates the CIEDE2000 by the formula: ∆E_00_ = (ΔL/K_L_ . S_L_) + (ΔC/K_C_ . S_C_)^2^ + (ΔH/K_H_ . S_H_)^2^ + R_T_ . (ΔC/K_C_ . S_C_) X (ΔH/K_H_ . S_H_)^0.5^

**Table 2:** Baseline L*, a* and b* mean values for specimens

	L*	a*	b*
Control	93.2	1,9	39.0
Saliva+EC	95.2	2.1	38.1
PHS+EC	95.9	2.0	37.2
Biosilicate+EC	94.7	2.0	38.1
PHS+Biosilicate+EC	96.0	1.9	37.3

Where, ΔL*, ΔC* e ΔH* are the differences in lightness, chroma, and hue between two measures and R_T_ (rotation function) is a function that accounts for the interaction between chroma and hue differences in the blue region. S_L_, S_C_ e S_H_ are the weighting functions for the lightness, chroma, and hue components. K_L_, K_C_ e K_H_ are the parametric factors according to different viewing parameters that were set to 1. 50:50% CIEDE 2000 perceptibility (50:50%PT = 0.8) and 50:50% acceptability (50:50%AT_00_ = 1.8) thresholds, standardized within ISO/TR 28642:2016, were used to analyze color differences in all the groups^36^ ^,^ ^37^.

### Microhardness Analysis

Three initial and final Knoop microhardness measures (HMV-2, Shimadzu Corporation, Kyoto, Japan) were obtained on each specimen with a vertical static load of 50 g for 5 s before and after the cycle procedure. As described for the surface roughness, the readouts were made at three defined locations, placing the sample with the help of the mark made earlier^29^. The change in microhardness was calculated relative to the initial measurement (%) using the formula ΔKHN = (final KHN - initial KHN)/initial KHN x 100.

### Surface roughness Analysis

Before and after the pH cycle procedure, surface roughness analysis was done using a roughness tester (Model SJ-201P Mitutoyo, Tokyo, Japan). The measures were taken at 3.2 mm distance and 0.8 mm cut-off at a speed of 0.5 mm/s. Three readings were taken as previously described to ensure measures at the same location before and after the protocols. The surface roughness alteration was calculated by the difference between the final and initial mean of these three readings.

### Statistical Analysis

Statistical analysis was performed using Prism version 9.3.1. Color stability (ΔE_00_), microhardness and surface roughness data were submitted to the Shapiro-Wilk normality test, which verified data distribution. The color stability and relative microhardness data were analyzed by One-way ANOVA and Tukey test (p < .05). The surface roughness data were compared by RM ANOVA and Tukey test (p < .05).

## Results

**Figure 2:**
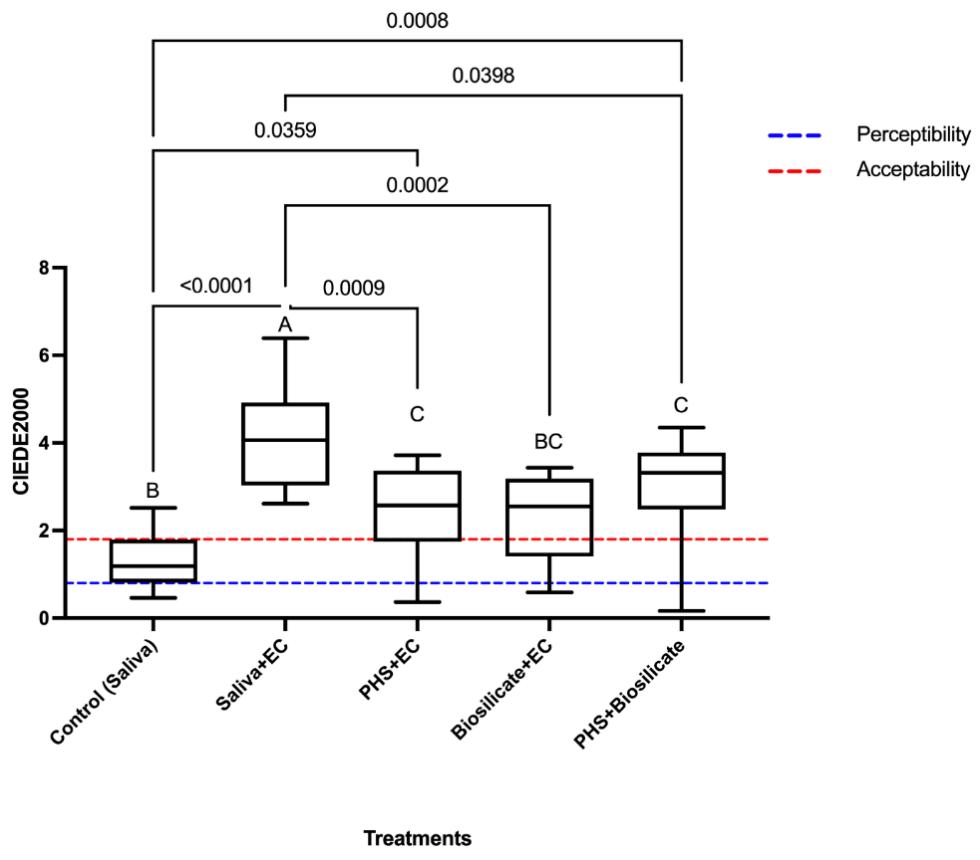
Means comparison of ΔE_00_ (One-way ANOVA, Tukey’s test, p<.05), and limits of perception (0.8) and acceptability (1.8). Different letters indicate statistically significant difference (p < .05)

The color alteration values were compared among the groups (Figure 2). The highest alteration occurred in the Saliva+EC group, different from all the other groups. The groups treated with PHS (PHS+EC and PHS+Biosilicate+EC) presented lower color change than the Saliva+EC group. The Biosilicate+EC group showed intermediate color stability, which was similar to all the other groups, except the Saliva+EC group. All the groups presented mean values above both the 50:50% perceptibility (50:50%PT = 0.8) and acceptability (50:50%AT = 1.8) thresholds, except for the control group (Saliva) that showed mean value above 50:50%PT but below 50:50%AT.

Table 3 compares the relative microhardness. The control group (Saliva) demonstrated the lowest alteration on the enamel microhardness, different (p < 0.0001) from all the other groups. The Biosilicate+EC group showed higher relative microhardness than the Saliva+EC group (p < 0.018), but similar to the PHS+EC and PHS+Biosilicate+EC groups, which presented intermediate values and were also similar to the Saliva+EC group.

Table 4 compares the surface roughness of the tested groups. The enamel surface roughness increased for all the groups. All the final values were statistically different from the initial ones (p < 0.0001), except for the control group that maintained its surface roughness (p > 0.999). The other groups revealed no difference among them (p > .05).

**Table 3:** Comparison of the relative microhardness mean values and standard deviations of the tested groups (One-Way ANOVA, Tukey’s test, p<.05).

Groups	Microhardness
Control	96.09 (14.39) A
Saliva+EC	31.30 (4.22) C
PHS+EC	41.15 (7.85) BC
Biosilicate+EC	44.21 (11.06) B
PHS+Biosilicate+EC	41.35 (8.80) BC

Different letters indicate statistically significant difference (p < .05).

**Table 4:** Comparison of the surface roughness mean values and standard deviations of the tested groups (Two-way ANOVA, repeated measures, p<.05).

	Control	Saliva+EC	PHS+EC	Biosilicate+EC	PHS+Biosilicate+EC
Initial	0.11 (0.02) aA	0.09 (0.02) aB	0.10 (0.03) aB	0.08 (0.02) aB	0.09 (0.02) aB
Final	0.12 (0.03) bA	0.33 (0.11) aA	0.33 (0.09) aA	0.31 (0.12) aA	0.27 (0.07) aA

Different letters, upper case in the column and lower case in the row, indicate statistically significant difference (p < .05).

## Discussion

This study evaluated the effect of phytosphingosine (PHS) and Biosilicate on the color stability, microhardness, and surface roughness of bovine dental enamel after erosive challenge (EC) with Coca-Cola®. There was change in color and microhardness in the group previously treated with the association of phytosphingosine and Biosilicate, so the null hypothesis was rejected.

Literature reports the effectiveness of fluoride solutions against demineralization and/or erosion, showing different results^20^ ^,^ ^38^ ^,^ ^39^. Furthermore, their efficacy depends on an intensive application^15^. In the present study, we proposed two novel treatments to protect the enamel: Using PHS solution and Biosilicate separately or in association. In addition, control groups were considered to compare the results.

Regarding color stability, both treatments exhibited less color alteration than the artificial saliva after erosive challenge with Coca-Cola (pH = 2.56). Beverages with low pH cause enamel color alteration^29^ ^,^ ^40^ and previous studies have demonstrated that the PHS solution protects the enamel against coffee (pH = 4.9) and black tea (pH = 5.4) similar to the effect of human saliva^29^ ^,^ ^40^. However, our findings were different probably because the samples were immersed in a more acidic solution. Acidic beverages can decrease the salivary pH affecting its buffer capacity^41^. Thus, the enamel is eroded and the dentin becomes more visible^13^ ^,^ ^14^.

On the other hand, even though the proposed treatments resulted in less color alteration, all the groups (expect for the control group) presented color alteration above the limit of acceptability (50:50%AT = 1.8)^36^, as observed by Amorim et al.^29^ after immersion in coffee and black tea.

It is important to assess the clinical significance of the color differences comparing the findings with the visual 50:50% perceptibility and acceptability thresholds. The results are irrelevant unless these parameters are used for clinically interpret the significant effect of the proposed treatments on the enamel color^42^.

A color difference at or below the 50:50% perceptibility threshold is desirable, but sometimes in clinical situations achieving a non-perceivable difference is difficult, costly and/or time-consuming. So, the 50:50% acceptable threshold is used to maintain the differences under an admissible limit^36^ ^,^ ^37^.

Thus, although the experimental treatments presented lower color alteration than the artificial saliva, the alteration is perceptible and not clinically admissible to the human eye.

Color change in all the groups also resulted from surface topographic alterations of the enamel due to decreased mineral content after EC^43^. A rougher surface gives more diffuse reflection and is more susceptible to staining, altering the perception of color^43^. The low pH of Coca-Cola causes change in roughness of the enamel that favors greater retention of stains^44^. Colas and other dark soft drinks have caramel color in their composition that promotes change in color, as noted in the present study^44^. All the groups presented higher enamel surface roughness after EC, except for the control group because it was not immersed in the acidic beverage. Apparently, the proposed treatments and the saliva were not able to prevent mineral loss induced by erosion.

As mentioned above, the PHS forms a protective layer over calcium hydroxyapatite that acts as diffusion barrier against H+ ions^3^ ^,^ ^22^; the saliva, for its part, would have several mechanisms involved in the protection of the enamel against erosion^12^; and the Biosilicate would be able to induce remineralization of the enamel^19^. However, evidently the protective mechanisms were not sufficient. After the erosive challenge, the enamel became significantly rougher with surface roughness values greater than 0.2 µm that according to previous studies not only alters the perception of color^43^, but also leads to biofilm accumulation^45^, increasing the risk of caries and periodontal inflammation^46^.

The most significant results of the present study are those related to enamel microhardness after EC. EC decreases the enamel microhardness due to dissolution of hydroxyapatite crystals^47^. Higher relative microhardness indicates that the treatment exhibited higher protective potential against demineralization^47^.

The group treated with Biosilicate demonstrated the highest relative microhardness among the experimental groups, significantly higher than the group immersed in saliva after EC. A previous study revealed that it would have a high potential preventing the mineral loss induced by dental erosion, showing higher microhardness than acidulated phosphate fluoride and the control group^48^. This can be justified by its basic pH (~ 9.0) after dissolution, and by its mechanism of action. It releases calcium and phosphate ions, and forms a silica-rich gel layer on mineralized tissues. The open structure of this layer allows the continuity of ionic exchange thus; an amorphous calcium phosphate layer is formed. Finally, it begins to crystallize into hydroxy(carbon)apatite^23^.

The groups treated with PHS, associated or not, showed similar results to the group treated with Biosiliate, revealing intermediate values. Therefore, apparently, the PHS did not contribute to prevent mineral loss. In a previous study, PHS presented less efficacy compared to toothpastes containing Sn^2+^ and F^-(^ ^22^.

Within the limitations of the study, it was concluded that the Biosilicate may prevent enamel mineral loss induced by erosion better than the saliva but has a limited action. The PHS did not enhance this effect but associated or not to Biosilicate demonstrated better color stability than saliva. Nevertheless, no treatment nor the saliva was effective in preventing enamel color alteration since the results were not clinically acceptable.

## References

[1] Gambon DL, Brand HS, Veerman ECI (2012). Dental erosion in the 21st century: What is happening to nutritional habits and lifestyle in our society?. Br Dent J.

[2] Rai N, Sandhu M, Sachdev V, Sharma R (2018). Evaluation of remineralization potential of beverages modified with casein phosphopeptide-amorphous calcium phosphate on primary and permanent enamel: A laser profiler study. Int J Clin Pediatr Dent.

[3] Valentijn-Benz M, Van ’t Hof W, Bikker FJ, Nazmi K, Brand HS, Sotres J (2015). Sphingoid bases inhibit acid-induced demineralization of hydroxyapatite. Caries Res.

[4] Bartlett DW, Lussi A, West NX, Bouchard P, Sanz M, Bourgeois D (2013). Prevalence of tooth wear on buccal and lingual surfaces and possible risk factors in young European adults. J Dent.

[5] Ali H, Tahmassebi JF (2014). The effects of smoothies on enamel erosion: An in situ study. Int J Paediatr Dent.

[6] Shahroom NS b, Balasubramaniam A, Nasim I (2020). Association Between Gender and Dental Erosion in South Indian Population. Eur J Mol Clin Med.

[7] Cheng R, Yang H, Shao M, Hu T, Zhou X (2009). Dental erosion and severe tooth decay related to soft drinks: A case report and literature review. J Zhejiang Univ Sci B.

[8] Li H, Zou Y, Ding G (2012). Dietary Factors Associated with Dental Erosion: A Meta-Analysis. PLoS One.

[9] Lussi A (2009). Dental erosion-Novel remineralizing agents in prevention or repair. Adv Dent Res.

[10] Carlos NR, Pinto AVD, Do Amaral FLB, França FMG, Turssi CP, Basting RT (2019). Influence of staining solutions on color change and enamel surface properties during at-home and in-office dental bleaching: An in situ study. Oper Dent.

[11] Karadas M, Seven N (2014). The effect of different drinks on tooth color after home bleaching. Eur J Dent.

[12] Baumann T, Kozik J, Lussi A, Carvalho TS (2016). Erosion protection conferred by whole human saliva, dialysed saliva, and artificial saliva. Sci Rep.

[13] Hanning M, Hanning C (2014). The pellicle and erosion. Monogr Oral Sci.

[14] Jager DHJ, Vieira AM, Ligtenberg AJM, Bronkhorst E, Huysmans MCDNJM, Vissink A (2011). Effect of salivary factors on the susceptibility of hydroxyapatite to early erosion. Caries Research.

[15] Philip N (2019). State of the Art Enamel Remineralization Systems: The Next Frontier in Caries Management. Caries Res.

[16] Ferreira JB, Paiva GR, Geraldo-Martins VR, Faraoni JJ, Dibb RGP, Lepri CP (2018). Influence of Remineralizing Dentifrice in the Treatment of Erosive Enamel Lesions. J Heal Sci.

[17] Neto FCR, Maeda FA, Turssi CP, Serra MC (2009). Potential agents to control enamel caries-like lesions. J Dent.

[18] Porcelli HBP, Maeda FA, Silva BR, Miranda WG, Cardoso PEC (2015). Remineralizing agents: Effects on acid-softened enamel. Gen Dent.

[19] Lepri CP, Macedo RP, Marra VF, Paiva GR, Castro DT de, Martins VRG (2020). Influence of Remineralizing Agents on the Surface Roughness of Eroded Dental Enamel: in Vitro Study. J Heal Sci.

[20] Ganss C, Klimek J, Brune V, Schürmann A (2004). Effects of two fluoridation measures on erosion progression in human enamel and dentine in situ. Caries Res.

[21] Bikker FJ, Hoogenkamp MA, Malhaoui A, Nazmi K, Neilands J, Krom BP (2018). Phytosphingosine prevents the formation of young salivary biofilms in vitro. Caries Res.

[22] Yönel N, Bikker FJ, Lagerweij MD, Kleverlaan CJ, van Loveren C, Özen B (2016). Anti-erosive effects of fluoride and phytosphingosine: An in vitro study. Eur J Oral Sci.

[23] Crovace MC, Souza MT, Chinaglia CR, Peitl O, Zanotto ED (2016). Biosilicate® - A multipurpose, highly bioactive glass-ceramic. In vitro, in vivo and clinical trials. J Non Cryst Solids.

[24] Pires-De-Souza FDCP, De Marco FF, Casemiro LA, Panzeri H (2007). Desensitizing bioactive agents improves bond strength of indirect resin-cemented restorations: Preliminary results. J Appl Oral Sci.

[25] Fanfoni L, Costantinides F, Berton F, Marchesi G, Polo L, Di Lenarda R (2020). From erosion to remineralization: The possible role of two topic home devices used as combined treatment. Appl Sci.

[26] Schlueter N, Hara A, Shellis RP, Ganss C (2011). Methods for the measurement and characterization of erosion in enamel and dentine. Caries Res.

[27] Mullan F, Austin RS, Parkinson CR, Hasan A, Bartlett DW (2017). Measurement of surface roughness changes of unpolished and polished enamel following erosion. PLoS One.

[28] Krikken JB, Zijp JR, Huysmans MCDNJM (2008). Monitoring dental erosion by colour measurement: An in vitro study. J Dent.

[29] Amorim AA, de Arruda CNF, Vivanco RG, Bikker F, de Pires-de-Souza FCP (2021). Effect of phytosphingosine on staining resistance and microhardness of tooth enamel. J Esthet Restor Dent.

[30] Huan L, Roeder LB, Lei L, Powers JM (2005). Effect of surface roughness on stain resistance of dental resin composites. J Esthet Restor Dent.

[31] Rios D, Ionta FQ, Rebelato R, Jordão MC, Wang L, Magalhães AC (2018). The effect of aspartame and pH changes on the erosive potential of cola drinks in bovine enamel: An in vitro study. J Clin Exp Dent.

[32] Hughes JA, West NX, Parker DM, Newcombe RG, Addy M (1999). Development and evaluation of a low erosive blackcurrant juice drink in vitro and in situ 1. Comparison with orange juice. J Dent.

[33] Devigus A, Lombardi G (2004). Shading Vita In-ceram YZ substructures: Influence on value and chroma, part II. Int J Comput Dent.

[34] Nigam AG, Murthy RC, Pandey RK (2009). Estimation of fluoride release from various dental materials in different media-An in vitro study. Int J Clin Pediatr Dent.

[35] Sharma G, Wu W, Dalal EN (2005). The CIEDE2000 color-difference formula: Implementation notes, supplementary test data, and mathematical observations. Color Res Appl.

[36] Paravina RD, Ghinea R, Herrera LJ, Bona AD, Igiel C, Linninger M (2015). Color difference thresholds in dentistry. J Esthet Restor Dent.

[37] International Organization for Standardization (2016). ISO/TR 28642; Dentistry-Guidance on Colour Measurement. ISO.

[38] Queiroz CS, Hara AT, Paes Leme AF, Cury JA (2008). pH-Cycling models to evaluate the effect of low fluoride dentifrice on enamel De- and remineralization. Braz Dent J.

[39] Mohammadi N, Farahmand Far MH (2018). Effect of fluoridated varnish and silver diamine fluoride on enamel demineralization resistance in primary dentition. J Indian Soc Pedod Prev Dent.

[40] Watts A, Addy M (2001). Tooth discolouration and staining: A review of the literature. Br Dent J.

[41] Mojaver YN, Javidi N, Manshaee K (2008). Influence of Soft Drink on Salivary pH. Chinese J Dent Res.

[42] Gasparik C, Manziuc MM, Burde AV, Ruiz-López J, Buduru S, Dudea D (2022). Masking Ability of Monolithic and Layered Zirconia Crowns on Discolored Substrates. Materials.

[43] Xiao B, Brainard DH (2006). Color perception of 3D objects: Constancy with respect to variation of surface gloss. Proc APGV.

[44] Chowdhury D, Mazumdar P, Desai P, Datta P (2020). Comparative evaluation of surface roughness and color stability of nanohybrid composite resin after periodic exposure to tea, coffee, and Coca-cola ” An in vitro profilometric and image analysis study. J Conserv Dent.

[45] Al Khuraif AAA (2014). An in vitro evaluation of wear and surface roughness of particulate filler composite resin after tooth brushing. Acta Odontol Scand.

[46] Lepri CP, Palma-Dibb RG (2012). Surface roughness and color change of a composite: Influence of beverages and brushing. Dent Mater J.

[47] Ávila DMDS, Augusto MG, Zanatta RF, Scaramucci T, Aoki IV, Torres CRG (2020). Enhancing the anti-erosive properties of fluoride and stannous with the polymer carbopol. Caries Res.

[48] Chinelatti MA, Tirapelli C, Corona SAM, Jasinevicius RG, Peitl O, Zanotto ED (2017). Effect of a bioactive glass ceramic on the control of enamel and dentin erosion lesions. Braz Dent J.

